# Development of a Decision Support System for the Management of Mummy Berry Disease in Northwestern Washington

**DOI:** 10.3390/plants11152043

**Published:** 2022-08-04

**Authors:** Mladen Cucak, Dalphy O. C. Harteveld, Lisa Wasko DeVetter, Tobin L. Peever, Rafael de Andrade Moral, Chakradhar Mattupalli

**Affiliations:** 1 Department of Plant Pathology and Environmental Microbiology, Pennsylvania State University, State College, PA 16801, USA; 2 Division of Biotechnology and Plant Health, Norwegian Institute of Bioeconomy Research (NIBIO), P.O. Box 115, NO-1431 Ås, Norway; 3 Department of Horticulture, Northwestern Washington Research and Extension Center, Washington State University, Mount Vernon, WA 98273, USA; 4 Department of Plant Pathology, Washington State University, Pullman, WA 99164, USA; 5 Department of Mathematics and Statistics, Maynooth University, W23 F2H6 Maynooth, Ireland; 6 Department of Plant Pathology, Northwestern Washington Research and Extension Center, Washington State University, Mount Vernon, WA 98273, USA

**Keywords:** *Monilinia vaccinii-corymbosi*, plant disease forecasting, decision support system, reproducibility, blueberry

## Abstract

Mummy berry, caused by *Monilinia vaccinii-corymbosi*, is the most important disease of the northern highbush blueberry (*Vaccinium corymbosum* L.) in North America and can cause up to 70% yield losses in affected fields. A key event in the mummy berry disease cycle is the primary infection phase where ascospores are released by apothecia that infect emerging floral and vegetative tissues. Current management of mummy berry disease in northwestern Washington is predominantly reliant on the prevention of primary infections through prophylactic, calendar-based fungicide spray applications early in the growing season. To improve the understanding of risk during these periods and to help tailor management strategies, we developed a decision support system (DSS) based on field records spanning over five seasons and four locations in northwestern Washington. Environmental conditions across the region were highly uniform but different dynamics of apothecial development were observed under high- and low-management regimes. Based on our analysis, we suggest basing the initial iteration of the DSS on two sub-models. The first sub-model predicts the onset of apothecia based on chill-unit accumulation under high- and low-management regimes, and the second predicts primary infection risk, which provides opportunities to improve the timing of fungicide applications. The synoptic DSS proposed here is based on the current biological knowledge of the pathosystem and available data for the northwestern Washington region. We provide the analysis and the DSS implementation and evaluation as an open-source repository, providing opportunities for further improvements. Finally, we provide suggestions for future research and the operational efforts needed for improving the utility and accuracy of the mummy berry DSS.

## 1. Introduction

Highbush blueberry (*Vaccinium corymbosum* L.; henceforth referred to as blueberry) is an economically important small fruit crop in North America, where approximately 91,400 acres are grown annually for fresh and processed markets in the USA (USDA NASS 2021). The Pacific Northwest (PNW) leads national production of blueberry in the USA with Washington and Oregon contributing 26% and 24%, respectively, of the national supply. One of the most important diseases of blueberry and other Vaccinium species in the region and elsewhere in North America is mummy berry, caused by the fungus *Monilinia vaccinii-corymbosi* (Reade) Honey (MVC) [1,2,3].

MVC infects its host in two phases. In the primary phase, overwintering pseudosclerotia produces apothecia that release ascospores (primary inoculum) to infect emerging tissues early in the growing season (primary infections) that develop as floral and vegetative strikes or ‘monilinia blight’. When infected, the entire floral cluster does not develop, causing significant losses in yield potential and defoliation [4]. In the secondary phase, conidia (secondary inoculum) produced on the strikes are disseminated to flowers by pollinators, primarily honeybees (*Apis mellifera* L.), via floral mimicry. Mycelial growth from germinated conidia infects developing ovaries (secondary infections), which eventually mummify the fruits that eventually drop off the plant [5]. Infected fruits cannot be consumed or processed. The mummified fruits or mummies turn into black, hard structures known as pseudosclerotia, which overwinter under the blueberry bushes. In early spring, apothecia develop from pseudosclerotia, producing ascospores that restart the disease cycle.

Mummy berry is an important disease of concern for blueberry growers in northwestern Washington [1,6], where the mild marine climate is conducive to the disease. The disease is mostly managed through intensive, calendar-based fungicide applications. Other methods such as mulching or apothecial removal can be effective but are often not feasible in large-scale operations. Apothecia releasing primary inoculum are the starting point of the disease cycle. If the crop can be protected from primary infections by fine-tuning the timing of fungicide applications applied during the primary infection phase, subsequent infections in the secondary phase can be prevented [7] and fungicide use decreased. Detection of apothecia in the field is laborious and time-consuming so a model indicating periods of high risk for apothecial presence would be useful to growers and crop consultants for making informed management decisions.

The timing of apothecial development and ascospore release is influenced by several environmental factors. Firstly, a chilling requirement of approximately 700 h below 7 °C is needed for pseudosclerotia to mature in Washington [1]. This differs among blueberry genotypes and local adaptation of the pathogen to different environments. For example, pseudosclerotia of rabbiteye blueberry (*V. virgatum* Aiton.) appear to be adapted to the low-chill environment in Georgia, USA, and needed ca 400–800 h [8], whereas lowbush blueberry (*V. angustifolium* Aiton.) in Maine, USA required 800–3000 h [9]. Secondly, the environmental factors identified to play a significant role in apothecia development and longevity are temperature, light, relative humidity, and soil moisture [3,7,10,11,12]. Lastly, the timing of apothecial development appears adapted to the timing of growth stages of early or late-maturing genotypes, indicating coevolution of the fungus with its host [13]. The host needs to be at a susceptible phenological stage for the initiation of disease. Vegetative tissues have been reported to be most susceptible when 6–13 mm of green tissue is present [4,7]. Floral tissues are considered most susceptible at the bud burst to tight cluster stages [4]. These factors all contribute to variation in apothecial development and the primary infection window, which complicates the development of a prediction model.

Several models have been described to estimate the timing of important events in the mummy berry disease cycle. Scherm [8] developed a model describing the relationship between cumulative chill hours and heating degree-days for germination of pseudosclerotia to aid in the timing of scouting and management of primary infections in rabbiteye blueberry in Georgia. A similar model was developed to estimate post-chill degree days needed for 25% of the maximum number of pseudosclerotia to produce apothecia in lowbush blueberry systems in Maine [9]. A preliminary model was developed to predict apothecial development for highbush blueberry in the PNW [12] but it has not been validated to date. The Mummy Berry Forecast System, developed by Delbridge and Hildebrand [14], is a method of applying curative fungicides within 72 h of infection periods, which are identified by air temperature and surface wetness. This forecast was tested in Maine for lowbush blueberries and was reported to be cost-effective and it produced timely information for growers, who now widely accept the system for disease control [15]. Combining knowledge from these existing models could help improve risk prediction [16], but this requires region-specific information and has not been done to date, especially for northwestern Washington.

Washington State University’s (WSU) AgWeatherNet (AWN) system offers a sustainable platform for providing weather data and decision support tools to growers, amongst which are plant disease risk models. While a risk prediction model for mummy berry is currently lacking, models are available to aid the management of other crop diseases such as grape powdery mildew caused by *Erysiphe necator* and hop powdery mildew caused by *Podosphaera macularis*. AWN is a university-based website accessible to the public, and hence provides an optimum location for the operational deployment of a mummy berry risk model, its maintenance, and further development.

The objective of this research was to develop a synoptic (mesoscale) decision support system (DSS) for the management of primary infections of the mummy berry disease by modeling the risk of primary infections based on available observations of apothecial presence and the set of environmental variables currently available on the AWN system. The outcomes of this research will help tailor mummy berry disease management strategies in northwestern Washington with timely protection of the crop and improve opportunities for economically and environmentally friendlier mummy berry management. Although some work was reported in Maine (USA) in the past, there is no full peer-reviewed report of a mummy berry DSS, nor is there any fully developed and operational DSS tool publicly available. Such a contribution provided by following open-science principles could aid further development of mummy berry disease risk prevention tools, both in Washington and worldwide.

## 2. Materials and Methods

In this paper, we initially provide a description of the regional weather conditions and their relationship to variation in the apothecial presence in space and time across the geographical domain. We then analyzed the available data to gain an understanding of the impact of different management levels on patterns of primary inoculum availability. This was done by means of statistical analysis as well as an estimation of thermal time required until the observance of the first apothecial presence (apothecial cups opened over 2 mm wide) in the field. To further improve the specificity of risk estimation for disease development, the inoculum availability model was then complemented with the infection risk model. The infection risk model was developed based on the most important environmental infection drivers available from the current literature. This enabled the estimation of the infection risk in terms of duration in hydrothermal time. Finally, we describe how the DSS was constructed, its visual presentation, and its evaluation with currently available data.

### 2.1. Data

**Figure 1:**
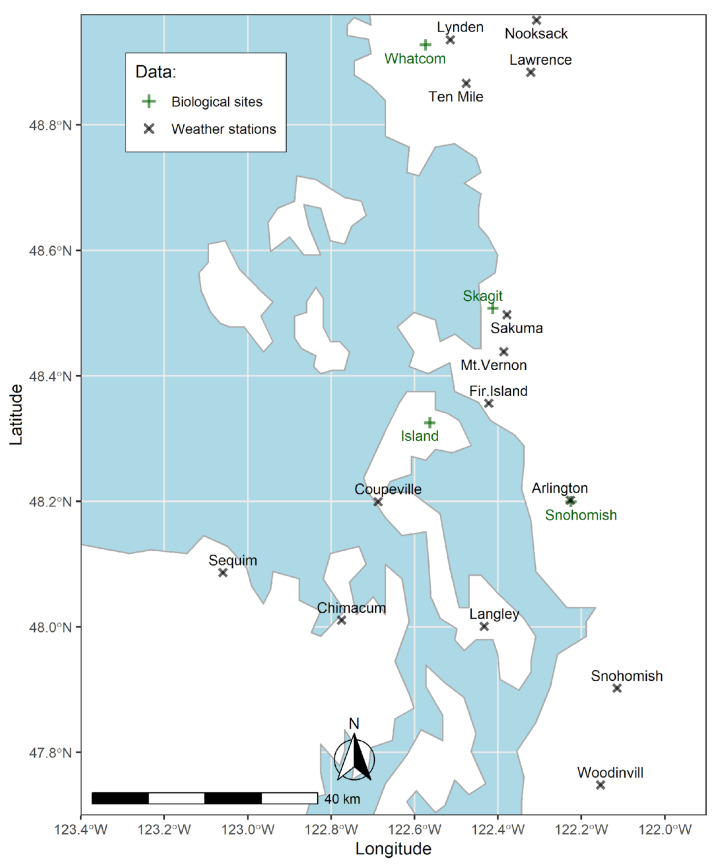
The geographical domain of the study area in northwestern Washington, USA. Green plus signs and black cross signs represent sites with biological data and locations of weather stations, respectively.

Biological and environmental data were collected from the northwestern region of Washington State (Figure 1).

#### 2.1.1. Biological Data

The apothecial development of MVC was monitored in four blueberry field sites in northwestern Washington between 2016–2019 and 2021 (Table 1). Monitored field sites included low and highly managed fields. In this research, low-disease management included no specific mummy berry management or minimal management, such as a single mulch application under the blueberry bushes without any other activity. High disease management consisted of active mulching and raking practices, including more than one mulch application and raking up to 2–3 times per week. The Skagit site changed to a different disease management regime after 2017 (Table 1), while other sites were consistent in their management practices. Observations of the apothecial development occurred on a weekly basis, typically from February until May and was repeated on a sample of 50–100 pseudosclerotia per site. During each assessment, pseudosclerotia spread across the field site were chosen arbitrarily and checked for pre-stages of apothecial formation. The state of the pseudosclerotium was recorded as germinating when a stipe or elongating tube was present or as apothecia when open cups were present. In this report, we use the term ‘apothecial presence’, which is the stage where the opening of the apothecial cup is larger than 2 mm and has the ability to release ascospores [11]. Apothecial presence was indicated as absence or presence (binary input) from the time that cups were open more than 2 mm wide until the cup presence disappeared.

#### 2.1.2. Environmental Data Collection

Raw weather data for the entire geographical domain and the historical period from 2000–2021, inclusive, were acquired from WSU’s AWN. The weather variables obtained included the hourly air temperature (℃) and relative humidity (%) at 2 m and the total hourly precipitation (mm) at 1 m. More information is available on the AWN webpage (www.weather.wsu.edu, accessed: 13 May 2022)).

**Figure 2:**
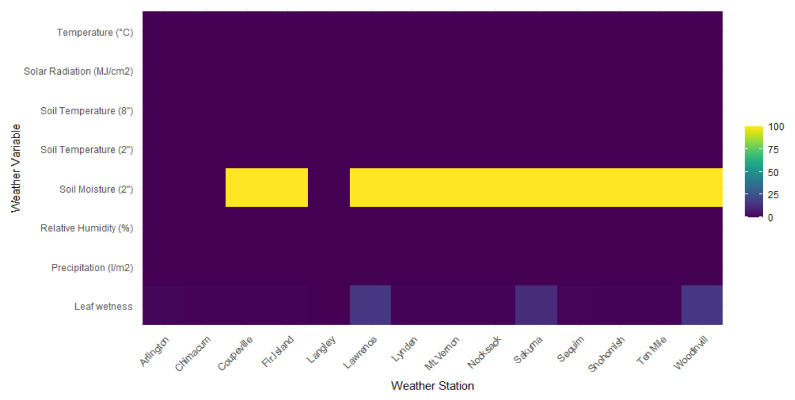
An overview of the available weather variables per weather station location with percentage of missing data (%). Data were obtained from Washington State University’s AgWeatherNet for the period 2000–2021.

The availability of the observed weather variables varied spatially (Figure 2).

#### 2.1.3. Data Processing

Data were provided in a 15-min resolution, which were then downscaled to an hourly resolution. Two data sets were then created, the first for the onset of apothecial presence and the second for initial evaluation of the entire DSS.

**Model development**. Each field site and season combination were considered an environment. The ‘haversine’ formula was used to calculate the great-circle distance between field sites and surrounding weather stations (Table 1). An exception to this was for the Island site, where the second closest station (Coupeville) was assigned because it was on the same island, compared to the closest station (Fir Island) located on the mainland and the difference in distance was less than 2 km. Each environment was assigned weather data from October of the preceding season until April of the season when the biological data were collected. The closest weather station with the entire time series, and less than 1% of missing data, was then found iteratively and assigned to each environment. The average distance between field sites and weather stations across seasons was 10.4 km (minimum of 9 and maximum of 12 km), which was considered fit for the intended purpose. This process resulted in a total of 15 environments.
**Model evaluation.** For the purpose of an initial evaluation, the weather data were split into station and year combinations, which were further reduced to those thatcontained no missing data. Finally, the data set for the model evaluation consisted of 134 environments.

### 2.2. Statistical Assessment of Characteristics of Apothecial Presence across Environments

**Figure 3:**
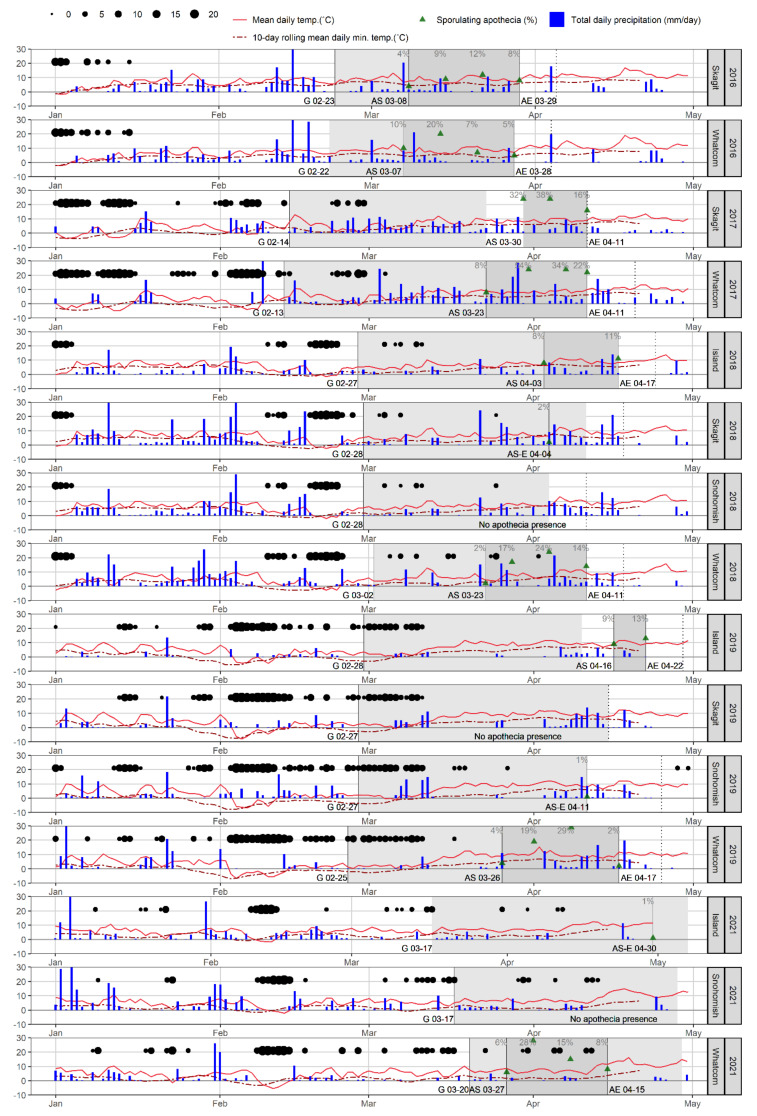
Daily weather and *Monilinia vaccinii-corymbosi* apothecial development records for the period 2016 to 2019 and 2021, at four sites (Island, Skagit, Snohomish, Whatcom (not present in all seasons)) in northwestern Washington. Daily weather conditions included average temperature and total daily precipitation (values over 30 mm not shown). Also included are the 10-day rolling daily mean minimum temperature (dashed line) and the sum of negative temperatures (<0 °C) in hours per day (represented with black dots, where the size of a dot corresponds to the total sum). Disease records: germination of pseudosclerotia, first and last observance of apothecia are represented by letters G (germination), AS (first observation of apothecial presence), AE (end observation of apothecia), or AS-E if on the same date followed by the corresponding date, respectively. Periods when germination and ascospore presence (open apothecia ready for sporulation) were observed are presented as light grey and grey areas, respectively. The proportion of apothecial presence is presented in dark green triangles.

To assess the impact of year, location, and management factors on the onset of apothecial presence, its intensity (the proportion of open apothecia), and duration, we fitted Cox proportional hazard models to the time-until-event data (onset and duration) and a gamma generalized linear model with a log link (intensity). We included the effects of year, management, and location as an additive in the linear predictors. The intensity of apothecial presence was measured as the area under the sporulation curve (AUSC). Here, AUSC was calculated following a trapezoidal method similar to the AUDPC method proposed by Madden et al. [17], as a sum of areas limited by time (in days) between the two assessments and the proportion of open apothecia on the two assessment dates (for reference see grey areas in Figure 3).

### 2.3. Prediction Models

Prediction models were developed for two life stages crucial for the timing of disease management practices. First, the accumulation of chill hours over time was used to estimate the probability of apothecial presence under the two management approaches, low- and high-management. Subsequently, a literature-based infection model by Hildebrand and Braun [4] was proposed to aid in identifying periods of high infection risk.

#### 2.3.1. Chilling Time to Apothecial Presence

Thermal time for the onset of apothecial presence was assessed using chill hours. Normally, biofix is determined based on the knowledge of the beginning of the winter period or provisional date, such as 1 January. The importance of this date is crucial in reducing the uncertainty of the initial date prediction. Hence, the accumulation of chill hours was initiated at five different (biofix) time points for each environment, 1 November, 15 November, 1 December, 15 December of the preceding year, and 1 January of the ‘current’ year. The accumulation was halted on the date when the presence of apothecia was first reported. Chill hours were accumulated as hours with a mean air temperature between 0 °C and 7.2 °C. Environments that did not have recorded apothecial presence were removed, leaving a dataset with 12 environments in total. The coefficient of variation was used to compare distributions obtained with different units, as follows:

(1) $$CV=\frac{\sigma }{\mu }$$

where *CV* stands for coefficient of variation, *σ* for standard deviation and *μ* for the population mean. Instead of relying on a crude threshold for indicating the initial date of apothecial presence, the cumulative probability of the initial apothecial presence was calculated over time based on the cumulative distribution function of normal distributions with means and variances obtained from each management level.

#### 2.3.2. Infection Model

Experimental data from Hildebrand and Braun [4] (Figure S1) were used to model the relationship between surface wetness duration, temperature, and disease severity. To ensure the operational functionality of the model, experimental data from Hildebrand and Braun [4] were extrapolated with an additional level of the temperature factor, which was beyond those in the original publication. This was done because the subset of the full weather data (as described in Section 2.1.2.) contained three hours with temperatures above 18 °C while surface wetness period conditions were met (rain ≥ 0.1 mm or RH ≥ 90%) in three different years. These three records demanded this extrapolation into the levels of the temperature factor, which were not tested, but were acceptable due to the low number of weather records. This was important not only for the reasons of operational application, but also for biological sensibility as although the data were not available beyond 18 °C, it is obvious that this is not a cardinal temperature for the pathogen activity.

Our extrapolated values correspond to the biological reasoning that the response of the pathogen is: (1) similar at the near-cardinal temperatures and (2) near-cardinal temperatures are equally distanced from the biological optimum. The optimum and minimum near-cardinal temperatures extracted from the data [4] were set at 16 °C and 2 °C, respectively. This is due to the fact that although the disease level was highest at 14 °C (Figure S1), disease levels were higher at 18 °C than at 10 °C on the two nearest temperature levels, indicating that the optimum is between 14 °C and 18 °C. The maximum near-cardinal temperature was assumed at 30 °C (i.e., 16 + 14) for which the observed disease severity at the opposite near-cardinal temperature (2 °C) was extrapolated.

The proportion of infected tissue as a function of hydrothermal time was then modeled using a beta model. A response surface over temperature and wetness duration was included in the linear predictor, with linear, quadratic, and cubic effects of temperature, and linear and log-linear effects of wetness duration. The significance of the effects was assessed using likelihood-ratio tests for nested models.

### 2.4. The Development of the DSS and Initial Evaluation

The DSS was constructed based on the two sub-models devised in Section 3 of this paper, in combination with existing mummy berry DSS schemes [15].

This DSS was evaluated with an existing weather dataset from the weather network (AgWeatherNet), which provides environmental data. The aim of such evaluation is normally two-fold. Firstly, to ensure that the model framework and parametrization are robust and correctly programmatically implemented, and that there are no nonsensical outputs. Secondly, the DSS was evaluated in terms of its ability to provide reasonable economic and environmental risk estimations. This evaluation was implemented using 134 environments that had no missing data for the variables required to run sub-models and was implemented in two segments:

(1) The apothecial presence onset date was calculated for all environments and the two management regimes. Bar charts with points corresponding to dates and descriptive statistics (quantiles) were created.
(2) The infection model was then activated after the onset of apothecial presence for a period of four weeks, assumed to be an approximate maximum duration of apothecial presence (as observed in our dataset, see Figure 3). Sums of days per each risk category and management regime were created and presented visually, including descriptive statistics (percentiles).

### 2.5. Data Analysis Software, Data, Code Availability, and Reproducibility

This entire analysis was implemented in the R (version 3.1) statistical programming language [18]. It can be partially reproduced using the repository archived at https://github.com/mladencucak/MBDSS (accessed: 13 May 2022). A portion of the data were imported using *readxl* [19]. The package *here* [20] was used to ensure reproducibility on different platforms. The package used for data munging was *tidyverse* [21]. The packages used for visualizations include: *ggplot2* [22], *ggthemes* [23], *plotly* [24] and maps were generated with package *sf* [25]. Univariate time series imputation of the weather data was implemented using functions from package *imputeTS* [26]. The package *lubridate* [27] was used for processing dates. Survival models were fitted using the *survival* package [28] and beta models were fitted with the *glmmTMB* package [29].

## 3. Results

### 3.1. Descriptive Analysis of Weather and Apothecia Data

The overall patterns of onset, duration, and intensity of apothecial presence during the studied period was consistent across sites and management levels, but not across environments (Figure 3 and Figure S2). The most striking observation was the similarity of weather conditions in each season, which was not matched by the expected apothecial presence across sites within seasons. While the apothecial presence was high and low, respectively, at the Whatcom and Snohomish sites across seasons, a trend of reduced apothecial presence was observed at the Island and Skagit sites over time (Figure S2).

During the study period, the initial day of apothecial presence at the Whatcom site was between 23 and 27 March except for an early start on 7 March in 2016. Onset, intensity, and duration of apothecial presence were similar at the Skagit and Whatcom sites in 2016 (Figure S2). This early apothecial presence occurred in a season with exceptionally warm winter conditions, as the 10-day rolling mean daily minimum temperature, accompanied by several rainy periods, was well above 0 °C from January onward. This pattern of similarity, in terms of apothecial presence between the two sites, was also observed in 2017, with the difference being that it had started somewhat later than in 2016. The pattern of apothecial presence at the Whatcom site remained similar during the following seasons (2018, 2019, and 2021). However, only two mature apothecia were found during the following two seasons (2018 and 2019) at the Skagit site. This abrupt reduction in apothecial presence coincided with the changes in production management from low- to high-management at the Skagit site (Table 1).

Weather conditions were remarkably similar across sites in different seasons (Figure 3). This specifically applies to the weather events and periods (such as the pattern of occurrence and the sum of daily negative temperatures, 10-day rolling daily mean minimum temperature, and rainy days) with specific importance for the development of apothecia from pseudosclerotia. Additionally, correlations of the mean daily temperature and wind, as well as cumulative rain across all stations in the geographical domain were very high (Figure S3). However, this pattern of similarity did not result in a consistent observance of apothecial presence at different sites, either across or within seasons. While apothecial presence was consistent during the study period at the Whatcom (low-managed) site, it varied significantly at high-management sites. The Whatcom site had a consistent four-week long duration of apothecial presence with higher intensity of apothecial presence. It should be noted that before the change in agronomic management practices, the Skagit site was similar to Whatcom in 2016 and 2017.

### 3.2. Statistical Assessment of Characteristics of Apothecial Presence Events across Environments

**Figure 4:**
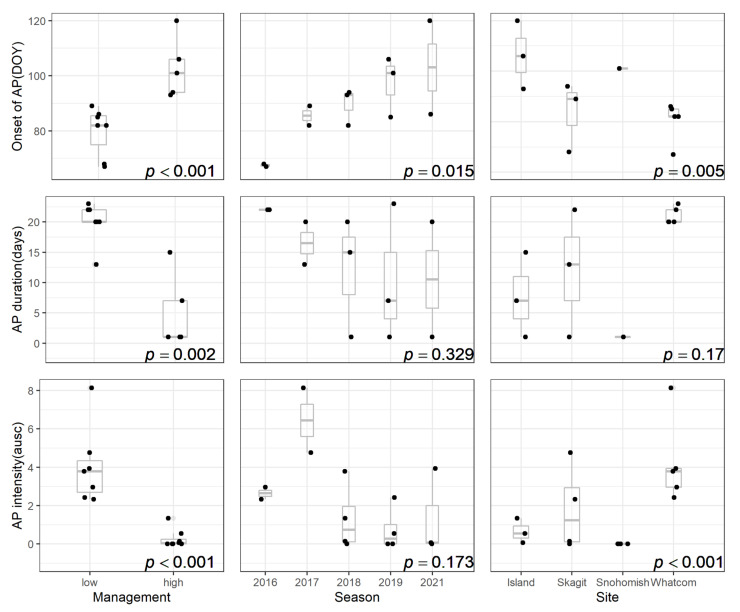
Statistical analysis of the impact of mummy berry disease management level, season, and site on the onset, duration, and intensity of apothecial presence (AP) in blueberry production systems in northwestern Washington. (DOY = Julian date; ausc = area under the sporulation curve).

Onset, duration, and intensity of apothecial presence were driven by the level of mummy berry management (Figure 4). The variable ‘season’ was only able to explain variability in the onset, and the variable ‘site’ significantly impacted the onset and intensity patterns.

### 3.3. Development of the DSS Sub-Components

#### 3.3.1. The Chilling Time Requirement for the Apothecial Presence

**Figure 5:**
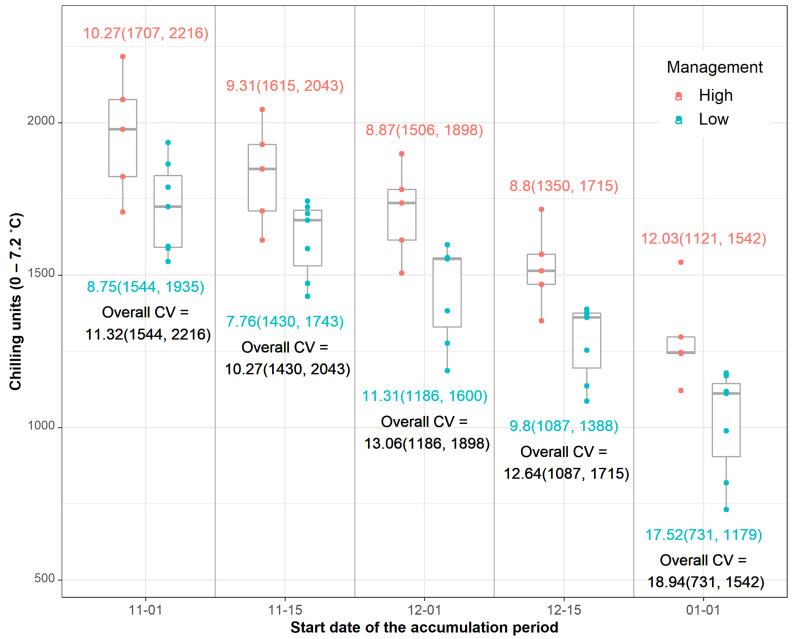
Accumulation of chill hours (hours with air temperatures between 0 and 7.2 °C) between varying biofix dates and the initial date of apothecial presence for each management level in blueberry production systems in northwestern Washington. Numeric values represent the coefficient of variation (CV) and minimum and maximum value (in brackets) per management level (color-coded) or overall (black color).

The optimum biofix date for initiation of the accumulation of the chilling hour time was determined after testing several initial dates. The overall coefficient of variation (CV) of the chill hour accumulation between varying biofix dates and the initial date of apothecial presence ranged from 10.27 to 18.94, for 15 November of the preceding year and 1 January of the current year, respectively (Figure 5). Furthermore, the lowest CV of 7.76 for low-management was recorded with the 15 November biofix and that of 8.87 for the 15 December biofix for high-management.

#### 3.3.2. The Infection Model

**Figure 6:**
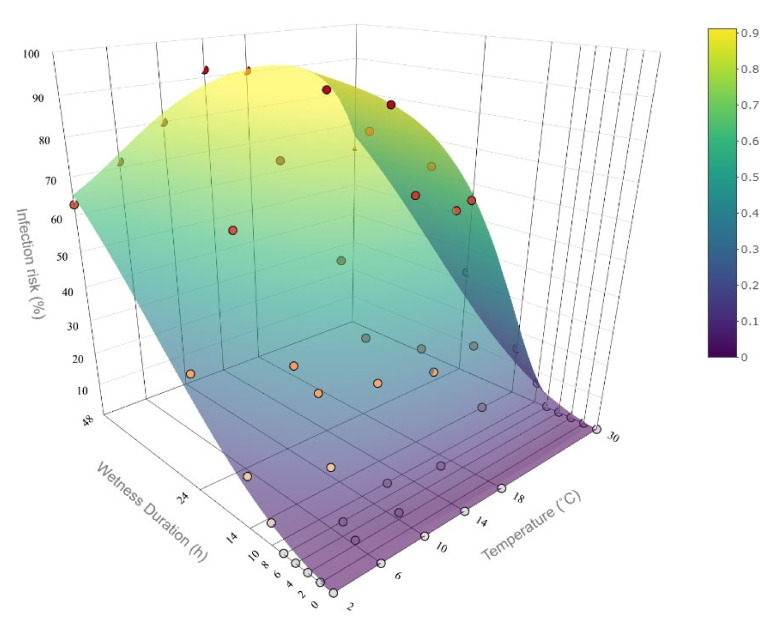
Surface plot representing the implementation of the *Monilinia vaccinii-corymbosi* apothecial presence model in northwestern Washington blueberry production systems fitted to experimental data by Hildebrandt and Braun [4] (represented with dots). The infection risk for the temperature at 30 °C was not present in the data set and was extrapolated. The surface represents the infection risk (%) modeled as a function of wetness duration and temperature.

The data set derived from the literature was used to model the intensity of the infection period based on the relationship between surface wetness duration and temperature (Figure 6). The proposed beta model with interactions provided a good fit both statistically (R^2^ = 0.928) and in terms of biological interpretation. The final model formula with coefficients is as follows:

(2) $$\begin{array}{ll} logit({\hat{\pi }}_{i})= & -7.08+2.06\times {temp}_{i}-1.02\times {temp}_{i}^{2}-6.63\times {temp}_{i}^{3}\\ & -0.03\times wetness_{i}+2.54\times loglog\;\left(wetness_{i}+1\right)\\ & +0.19\times temp_{i}\times wetness_{i}+0.38\times temp_{i}^{2}\times wetness_{i}\\ & -0.14\times temp_{i}^{3}\times wetness_{i}-3.12\times temp_{i}\times log\;(wetness_{i}+1)\\ & -5.62\times temp_{i}^{2}\times log\;(wetness_{i}+1)\\ & +3.18\times temp_{i}^{3}\times log\;(wetness_{i}+1) \end{array}$$

where *temp* stands for temperature (°C) and *wetness* for surface wetness duration (h).

The data and corresponding model fit indicate the characteristic S-shaped increase in the level of plant disease infection with an increase in wetness duration. More specifically, there is a steep increase in infection risk after about 8–9 h of wetness duration especially at temperatures near optimum. The rate of the infection risk increase levels off after about 20 h.

### 3.4. Implementation and Initial DSS Evaluation

**Figure 7:**
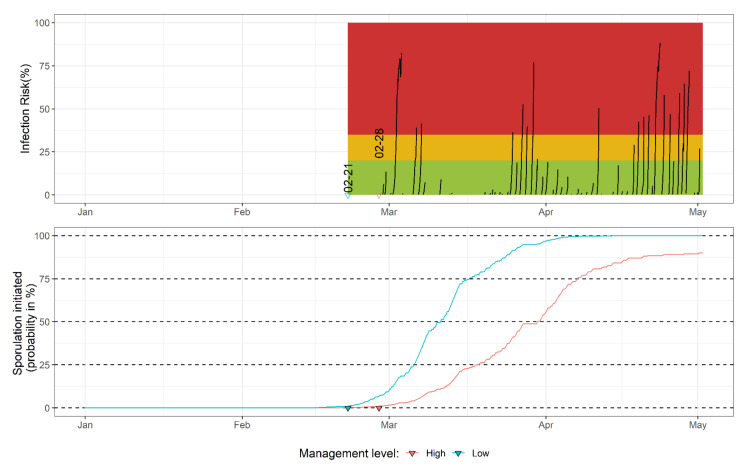
The mummy berry DSS developed in northwestern Washington blueberry fields with low- or high-management levels for the disease. The upper graph represents the percentage of infection risk, where the green, orange and red areas represent the low, medium and high level of risk, respectively. The dates (formatted as: month-day) above triangles correspond to a 1% probability of apothecial presence in the field in two management systems. The lower graph presents the probability of apothecial presence.

The system consists of two sub-models, the prediction of the onset of apothecial presence over two distinct management regimes, and the infection model (Figure 7). The probability of apothecial presence is estimated based on the accumulation of chill hours from 15 November of the previous year (see Section 3.3.1). The infection model is activated once it reaches the threshold of a 1% probability of apothecial presence in the low-management regime. The infection risk is presented as a percentage to aid user perception (see Figure 6). The surface wetness was estimated using a traditional approach proven to give satisfactory results on several crops [30,31,32], where the plant surface is assumed to have a high chance of being wet when rain ≥ 0.1 mm or RH ≥ 90%. The maximum break in infection conditions that would not stop the infection sub-model accumulation was set to two hours [33].

The traffic light-based interpretation of risk estimation is extrapolated from the mummy berry DSS used in northeastern parts of North America [14]. The application of fungicides is recommended after the model indicates high infection risks (infection risk: >35%, red area on the upper graph in Figure 7). The yellow area (infection risk: 20–35%) indicates the medium risk level and suggests caution. In practice, fungicide treatment is recommended when several such events occur within a short period of time or even a single instance if there is susceptible tissue present and protective fungicide has been washed off, warranting fungicide application. The green area represents periods with a minimum to no infection risk (infection risk: <20%). The risk of infection only exists if there is susceptible tissue available on the host [4,7]. Producers are encouraged to monitor blueberry phenological stages and consult regular mummy berry updates provided by WSU (archived example available at: https://extension.wsu.edu/berrypathology/mummyberry-update/, accessed: 13 May 2022). Monitoring of the apothecial presence is an ongoing effort, and the current sporulation model parametrization will be re-evaluated on a yearly basis. The model implementation code in the R language provided in our analysis is used in the AWN system, which allows yearly re-parameterization.

**Figure 8:**
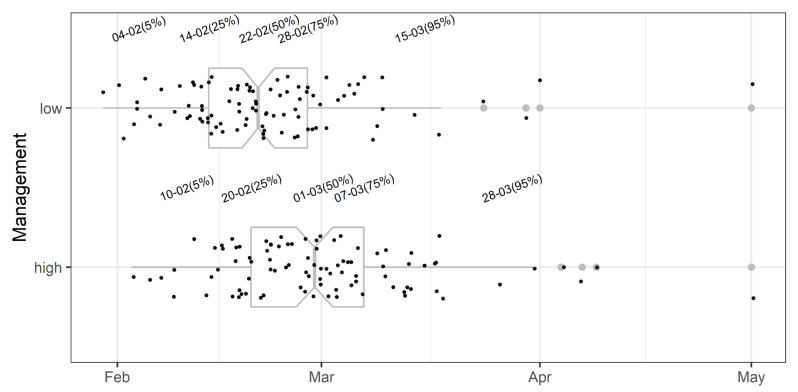
Initial evaluation of the *Monilinia vaccinii-corymbosi* apothecial presence sub-model in blueberry production systems in northwestern Washington. Points represent the date when the probability of 1% apothecial presence was calculated for low and high management levels in each of 134 environments. Grey boxplots represent the cutoff dates at 5, 25, 50, 75, and 95% across environments, and corresponding grey dots represent the outlier values. Dates (formatted as the day of the month followed by month) above box plots correspond to percentiles of the entire data set for each management level.

Initial evaluation of the prediction model showed that the mean predicted onset of apothecial presence is on 22 February and 1 March under low- and high-management regimes, respectively (Figure 8). Half of the predictions for the initial onset of apothecial presence were within a two-week period between 14 February and 28 February, and between 20 February and 7 March, under the low- and high-management regimes, respectively. There were four occurrences above the highest percentile, and all of them occurred in 2010.

**Figure 9:**
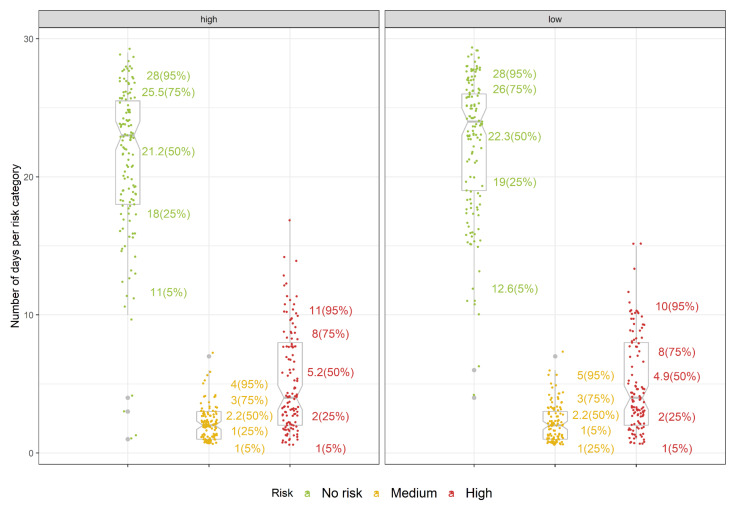
Initial evaluation of the mummy berry infection sub-model for blueberry in northwestern Washington. Individual points represent the number of days classified in the no-risk, medium and high-risk categories out of the four-week evaluation period, for high(left) and low(right) management levels. The numbers on the side of the grey box plots correspond to percentiles of the total sum per risk category. Grey dots represent the outlier values.

Evaluation of the infection model showed that on average, 21.2 and 22.3 days have indicated no risk, 2.2 in both cases have indicated medium risk and 5.2 and 4.9 indicated high risk under high- and low-management regimes, respectively (Figure 9). Mean sums of 5.2 and 4.9 days during the four-week evaluation period (assumed to be a maximum duration of apothecial presence) for high- and low-management regimes, respectively, indicate that the infection model has the potential to be a useful tool in guiding the timing and if conditions allow, extending the periods between the fungicide applications.

## 4. Discussion

This report describes the development and operational deployment of a web-based DSS to aid mummy berry disease management for highbush blueberry in northwestern Washington. This was accomplished by combining our data with currently available knowledge of mummy berry epidemiology and a prediction model developed for lowbush blueberries in northeastern United States. To facilitate further development of mummy berry DSS, the entire analysis, model implementation code, and biological and partial weather data are publicly available. The test version of the model is available for registered users (free registration) through Washington State University’s AgWeatherNet system (https://weather.wsu.edu/, accessed: 13 May 2022).

Different patterns of duration and intensity of apothecial presence were observed in northwestern Washington field sites across growing seasons despite the similarity of synoptic weather conditions across sites within growing seasons. Similar to previous reports [7,12,34,35,36,37], apothecial presence was observed for three to four weeks at the low-management sites between February to May. While these periods have been consistently high and low for apothecial presence at the Whatcom and Snohomish sites, respectively, a trend of reduction was observed at the Island and Skagit sites. We speculate that information from our data collection and elevated grower awareness that open apothecia were present may have led to more intensive management of pseudosclerotia at specific sites.

Burying the pseudosclerotia in early spring has been shown to suppress apothecial development [38,39]. Raking or disruption of pseudosclerotia disturbs their development by changing their local environment as well as by damaging developed apothecia [3]. These disease management practices were implemented in highly managed sites in our study, which led to reductions in apothecial presence. The results of our study align with these previous reports, as the level of management was inversely proportional to apothecial presence (see Figure 3). Epidemiologically, the control of primary infections leads to reductions in the rate of secondary infections, and causally, a reduction in primary inoculum the following season. Several factors drive these reductions, such as the disease management practices, the mode of action of fungicides used for disease control, and fungicide rotation within and between seasons, which impact the selection patterns within the pathogen population. These segments appear not well understood and under-represented in the literature and there is little information about the current population diversity of *M. vaccinii-corymbosi,* especially from the PNW [40]. Lack of such information could explain why despite fewer open apothecia, diseased shoot and floral strikes were still observed within these sites (data not presented). Additionally, although apothecial presence was reduced, a single apothecium is estimated to produce ~61.5 × 10^6^ ascospores [11]. Therefore, a single apothecium exerts disease pressure and is a reminder that users of the DSS should exercise caution in interpreting levels of risk for disease development.

The DSS comprises two sub-components. The first sub-component provides a probability estimation of the onset of apothecial presence under low- and high-management regimes to provide an indication of the onset of sporulation periods and assist scouting efforts. Our analysis has shown that the level of disease management was a major limiting factor of apothecial development and that it should be factored into the prediction of onset of apothecial presence. Apothecial development from pseudosclerotia occurs in several stages from germination to open apothecia [41]. There is a lack of agreement in the literature with regard to environmental conditions required for carpogenic germination, although several authors report the importance of chill hours during the winter period [9,10,11,39]. This might be due to several factors originating from differences in measurement accuracies, features of agro-climatic zones, and/or host biological, phenological, or morphological nature (e.g., blueberry species) that influence the interaction between host and pathogen. It should be noted that chill hours are calculated from different start dates, although this information is sometimes not even reported. Therefore, we found an optimal biofix by testing several dates from which to start the accumulation of chill hours. The variability of chill hours necessary for the onset of apothecial presence was lowest if the biofix for their accumulation was set on 15 November. From the perspective of the host plant, the period shortly after 15 November is when the plant is likely still acclimating to winter conditions depending on seasonal conditions [42,43].

The second sub-component is an infection risk sub-component, which will aid in the tailoring of fungicide management programs. Intensive disease management in blueberry fields greatly reduces primary inoculum, which has the potential to significantly affect the rate of mummy berry epidemics [4]. Besides the operational in-season tactical value, the use of this tool will contribute to the general education of growers [44], leading to a better understanding of the relationship between weather patterns favorable for increased disease pressure.

This iteration of the mummy berry DSS reflects current scientific knowledge of the epidemiology of the mummy berry disease and operational constraints (e.g., availability of in situ soil and atmospheric environmental data). For example, we had to extrapolate the available data for the expected disease levels at temperatures above 18 °C to allow and ensure the operational applicability of this sub-component of the DSS. Environmental factors such as temperature, relative humidity, light, and soil moisture influence apothecial development and longevity [3,7,10,11,12]. In particular, the hydrothermal state of the top layer of the soil appears to be a key driver of carpogenic development of apothecia [12], and these conditions are approximated using air measurements that served as proxy variables. However, the development of a synoptic model with the aim of deployment on a certain weather network is limited to the environmental variables measured by the network in question.

This DSS provided as an open-source prediction tool should also help seasonal re-parametrization and upgrades in terms of modeling approaches based on ongoing seasonal data collection efforts, developing scientific knowledge, and expanding databases. An additional benefit is this open-source prediction tool is available for external evaluation, validation, and continued refinement. While the initial implementation of this DSS should provide a useful tool, spaces for improvement have been identified. Hence, our approach is to continue yearly re-parametrization of the model based on ongoing data collection efforts. The model presented here is an initial effort to establish a foundational platform for further upgrades by the community of other researchers working on this important disease.

## Figures and Tables

**Table 1:** Field sites in northwestern Washington monitored for *Monilinia vaccinii-corymbosi* apothecia during the 2016–2019 and 2021 study periods. Data were not collected during the 2020 growing season.

Growing Season	Site Location (County)	Disease Management	Weather Station	Distance of Field Site from Weather Station (km)
2016	Skagit	Low	Sakuma	9
	Whatcom	Low	Lynden	10
2017	Skagit	Low	Sakuma	9
	Whatcom	Low	Lynden	10
2018	Skagit	High	Sakuma	9
	Whatcom	Low	Lynden	10
	Island	High	Coupeville	12
	Snohomish	High	Fir Island	12
2019	Skagit	High	Sakuma	9
	Whatcom	Low	Lynden	10
	Island	High	Coupeville	12
	Snohomish	High	Arlington	10
2021	Whatcom	Low	Ten Mile	12
	Island	High	Coupeville	12
	Snohomish	High	Langley	14

## Data Availability

Repository available at https://github.com/mladencucak/MBDSS accessed: 13 May 2022.

## Supplementary Materials

The following supporting information can be downloaded at: https://www.mdpi.com/article/10.3390/plants11152043/s1, Figure S1: Experimental data by Hildebrand and Braun [4] used for the infection model development. Figure shows the effect of different temperatures and wetness duration on disease severity; Figure S2: Daily weather and *Monilinia vaccinii-corymbosi* apothecial development records for the period 2016 to 2019 and 2021, at four sites [Island, Skagit, Snohomish, Whatcom (not present in all seasons)] in northwestern Washington. Daily weather conditions included average temperature and total daily precipitation (values over 30 mm not shown). Also included are the 10-day rolling daily mean minimum temperature (dashed line) and the sum of negative temperatures (<0 °C) in hours per day (represented with black dots, where the size of a dot corresponds to the total sum). Disease records: Germination of pseudosclerotia, first and last observance of apothecia are represented by letters G (Germination), AS (first observation of apothecia presence), AE (End observation of apothecia), or AS-E if on the same date followed by the corresponding date, respectively. Periods when germination and ascospore presence (open apothecia ready for sporulation) were observed are presented as light grey and grey areas, respectively. The proportion of apothecial presence is presented in dark green triangles; Figure S3: Weather correlations are presented using correlograms. Weather stations have been ordered so that they reflect corresponding latitudes (e.g., Woodinville is northernmost weather station). Correlograms correspond to the following variables: (a) all variables together, (b) relative humidity, (c) temperature, (d) rain. The three stars symbol above numbers in correlograms represents high level of significance.
