# Supplementary material for: Development of a Decision Support System for the Management of Mummy Berry Disease in Northwestern Washington
Source: Plants (Basel). 2022 Aug 4;11(15):2043. doi: 10.3390/plants11152043 (PMC9370572; doi:10.3390/plants11152043)
Supplement: Supplementary file 1 [file plants-11-02043-s001.zip › plants-1748164-supplementary.pdf]

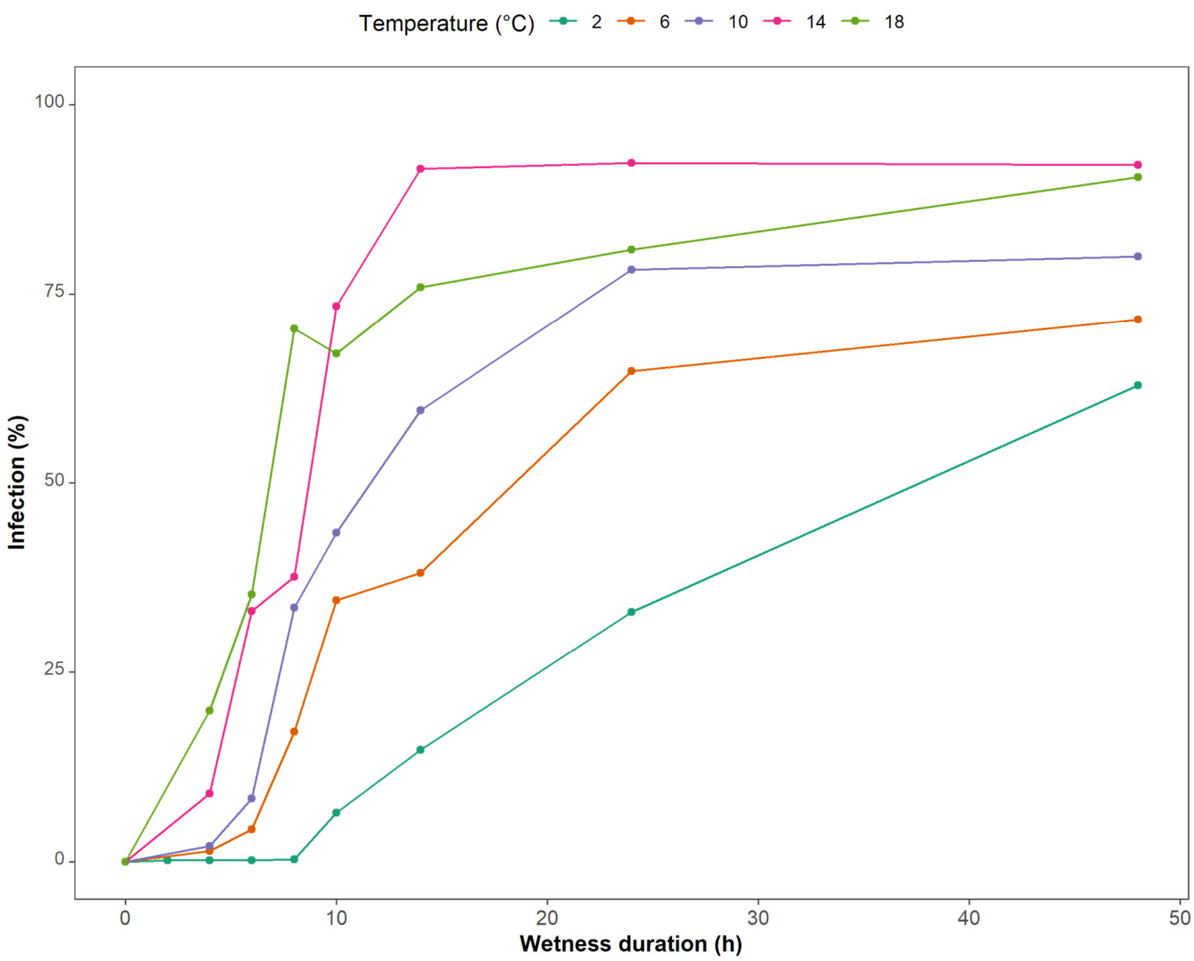

Figure S1. Experimental data by Hildebrand and Braun [4] used for the infection model development. Figure shows the effect of different temperatures and wetness duration on disease severity.

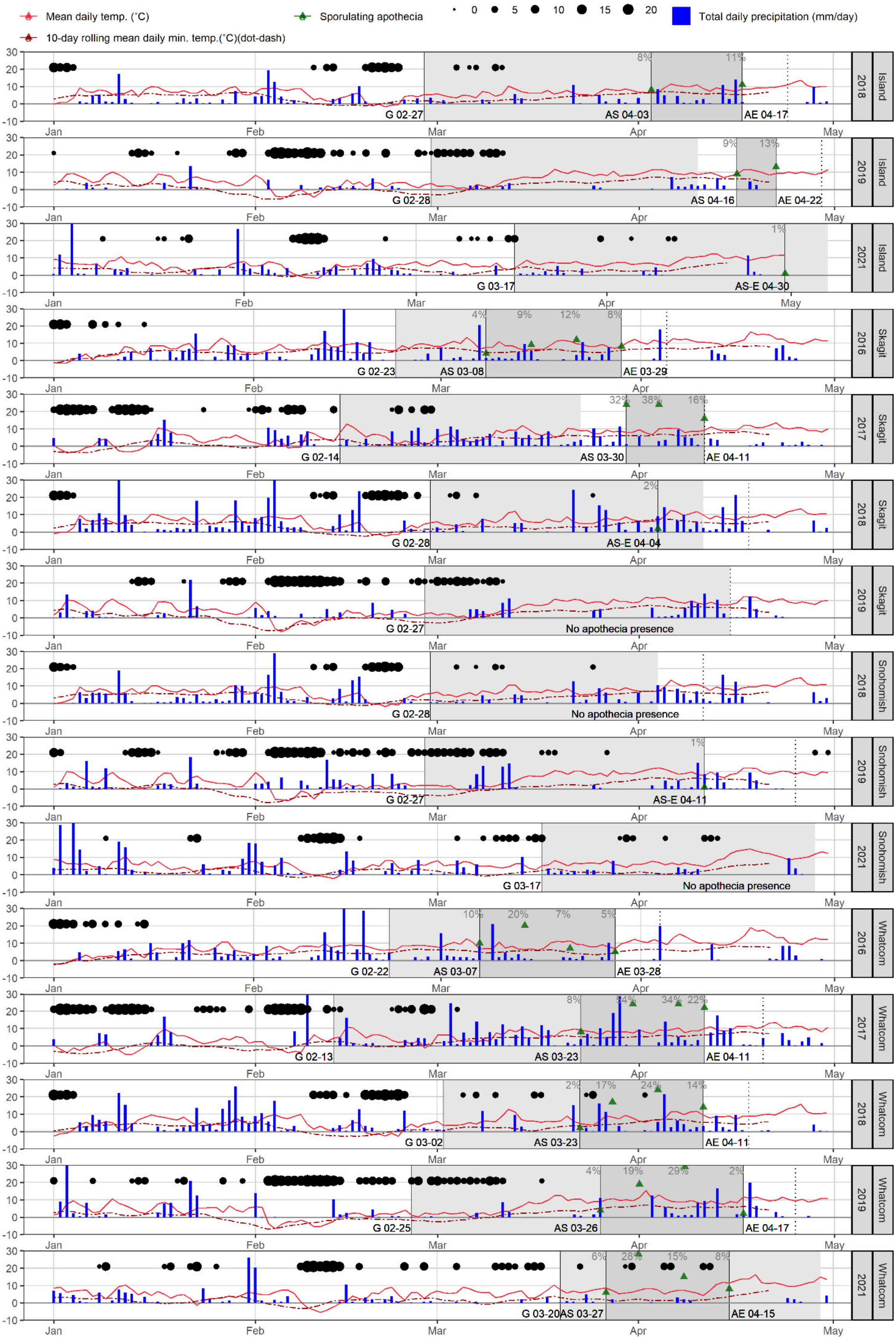

Figure S2. Daily weather and *Monilinia vaccinii-corymbosi* apothecial development records for the period 2016 to 2019 and 2021, at four sites [Island, Skagit, Snohomish, Whatcom (not present in all seasons)] in northwestern Washington. Daily weather conditions included average temperature and

total daily precipitation (values over 30 mm not shown). Also included are the 10-day rolling daily mean minimum temperature (dashed line) and the sum of negative temperatures (< 0 ˚C) in hours per day (represented with black dots, where the size of a dot corresponds to the total sum). Disease records: Germination of pseudosclerotia, first and last observance of apothecia are represented by letters G (Germination), AS (first observation of apothecia presence), AE (End observation of apothecia), or AS-E if on the same date followed by the corresponding date, respectively. Periods when germination and ascospore presence (open apothecia ready for sporulation) were observed are presented as light grey and grey areas, respectively. The proportion of apothecial presence is presented in dark green triangles.

a)

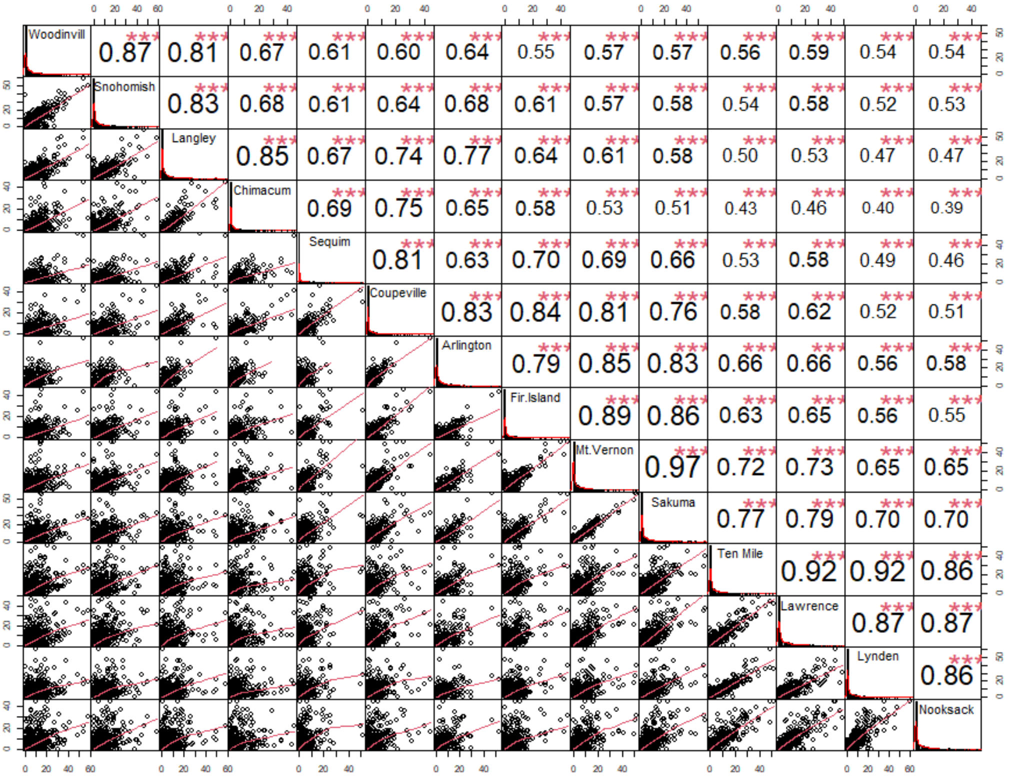

b)

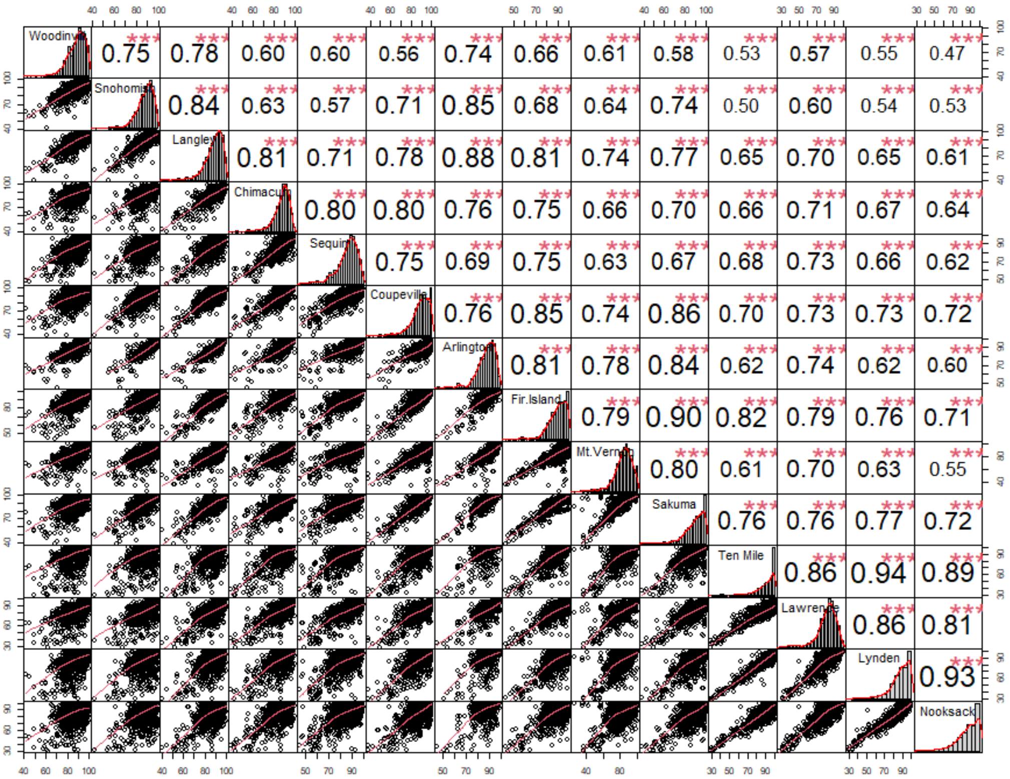
Woodinv
Snohomis
Langley
Chimacu
Sequi
Coupevill
Arlingto
Fir.Island
Mt.Vern
Sakuma
Ten Mile
Lawrence
Lynden
Nooksack

c)

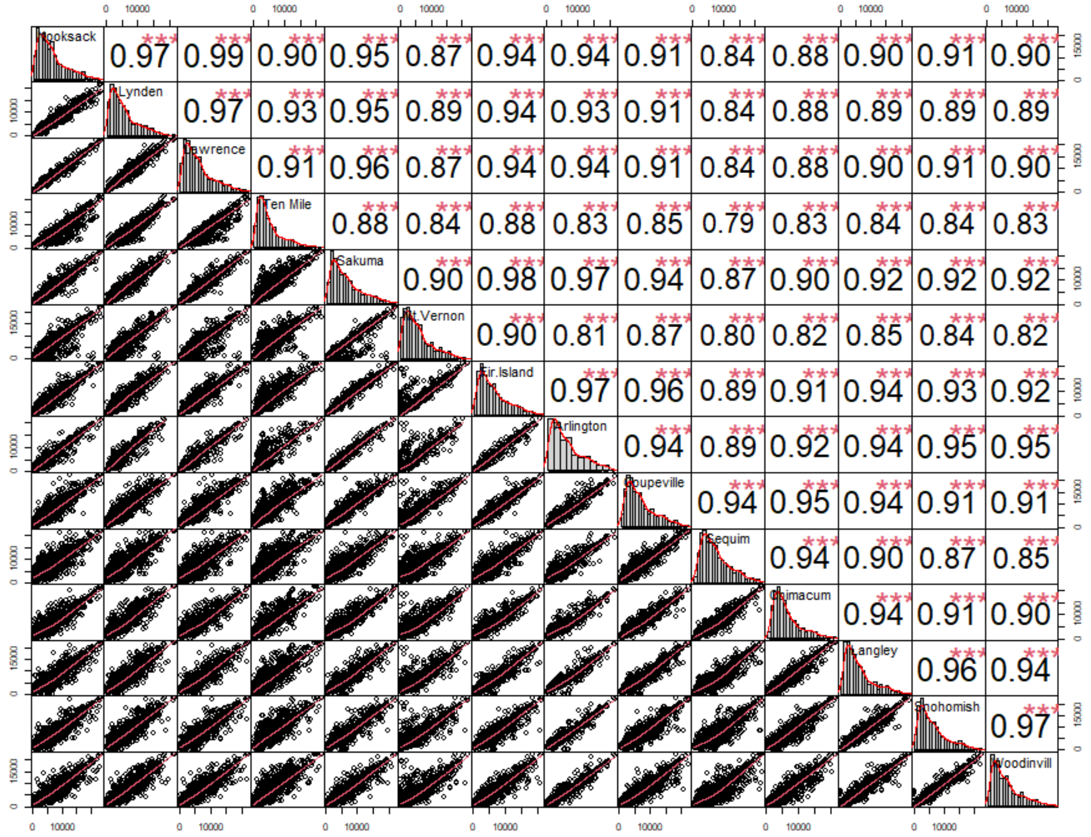
ooksack
Lynden
awrence
Ten Mile
Sakuma
t.Vernon
ir.Island
rlington
oupeville
equim
imacum
angley
nohomish
Woodinvill

d)

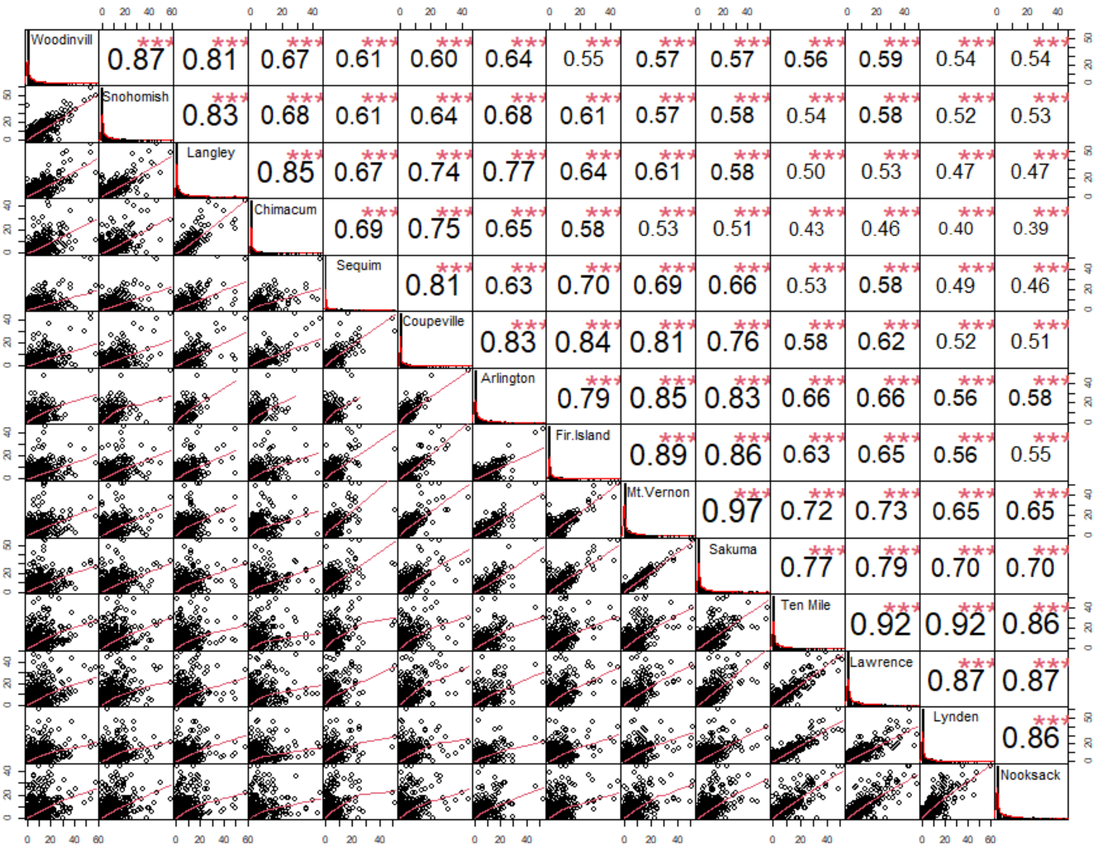

Figure S3. Weather correlations are presented using correlograms. Weather stations have been ordered so that they reflect corresponding latitudes (e.g., Woodinville is the northernmost weather station). Correlograms correspond to the following variables: a) all variables together, b) relative humidity, c) temperature, d) rain.The three stars symbol above numbers in correlograms represents high level of significance.
